# 儿童原发性纯红细胞白血病3例报告并文献复习

**DOI:** 10.3760/cma.j.cn121090-20251109-00515-1

**Published:** 2026-06

**Authors:** 笛箫 钟, 娟娟 李, 蕾 张, 朝霞 张, 君惠 李, 梦泽 胡, 嵘 刘

**Affiliations:** 首都医科大学附属首都儿童医学中心，北京 100020 Capital Center for Children's Health, Capital Medical University, Beijing 100020

## Abstract

本研究旨在探讨儿童原发性纯红细胞白血病（PEL）的临床特征，尤其NFIA::CBFA2T3和NFIA::RUNX1T1融合基因阳性PEL的临床特点。回顾性分析2017年9月至2021年9月在首都医科大学附属首都儿童医学中心收治的3例儿童PEL的临床资料，并进行文献复习。3例患儿均检测到细胞遗传学改变，例1检出t（1;16）（p32;q24），例2检出NFIA::CBFA2T3融合基因，例3检出NFIA::RUNX1T1融合基因。3例患儿对急性髓系白血病（AML）标准强化化疗及含有去甲基化药物的化疗均反应不佳，例1、3疾病持续进展死亡，例2行强化疗桥接脐血干细胞移植，移植后2个月复发，后迅速死亡。本研究提示儿童原发性PEL具有起病隐匿、进展迅速、对AML常用化疗方案不敏感的临床特征，在完全缓解后行造血干细胞移植可能获益。

## Abstract (en)

This study aimed to investigate the clinical characteristics of primary pure erythroid leukemia（PEL）in children, particularly PEL with NFIA::CBFA2T3 or NFIA::RUNX1T1 fusions. We retrospectively analyzed clinical data from three children with PEL who were treated at the Capital Center for Children's Health, Capital Medical University, between September 2017 and September 2021, and reviewed the literature. All three patients had cytogenetic alterations: case 1 had t（1;16）（p32;q24）, case 2 had an NFIA::CBFA2T3 fusion, and case 3 had an NFIA::RUNX1T1 fusion. All three patients responded poorly to standard intensive chemotherapy for acute myeloid leukemia（AML）and to regimens containing hypomethylating agents. Cases 1 and 3 died after continued disease progression. Case 2 underwent intensive chemotherapy followed by umbilical cord blood stem cell transplantation, relapsed 2 months after transplantation, and died soon thereafter. These findings suggest that primary PEL in children is characterized by an insidious onset, rapid progression, and poor response to commonly used AML chemotherapy regimens, and that patients may benefit from hematopoietic stem cell transplantation after achieving complete remission.

纯红细胞白血病（Pure erythroid leukemia, PEL）是一种罕见类型的急性髓系白血病（AML），以红系前体细胞增殖伴分化阻滞为主要特点，起病隐匿，进展迅速，对常规化疗不敏感，预后极差。由于红系肿瘤的特殊性，也易出现诊断延误。我们报告近年来我院收治的3例儿童原发性PEL患者的临床特点及诊疗经过，并进行文献复习，旨在提高对儿童原发性PEL的认识。

## 病例资料

例1，女，7月龄，因“体检发现血红蛋白水平下降半个月”于2017年11月入院。院外查血常规：HGB 81 g/L，WBC 15×10^9^/L，分类正常，PLT正常，外周血涂片见4％～7％异常淋巴细胞。腹部超声示脾大，予补铁治疗，效果不佳。复查血常规WBC 31.34×10^9^/L，HGB 59 g/L，PLT正常，外周血涂片见12％原始幼稚细胞，LDH 32 940 U/L。院外骨髓穿刺涂片：增生活跃，粒∶红为0.08∶1，红系明显增多，占88％，倾向于原始、早幼红细胞。既往史、个人史及家族史无异常。体检：面色苍白，肝肋缘下10 cm，脾肋缘下5 cm。我院骨髓流式细胞术（FCM）分析：CD45阴性且SSC较大的分布区域可见异常细胞群体，约占有核细胞的33.9％，表达CD36、CD117，部分细胞表达CD71^dim^、GlyA，少数细胞表达CD7；核型：46,XX,t（1;16）（p32;q24）[12]/46,XX[8]。骨髓基因突变：KRAS: NM_004985，exon3，c.175G>A（p.A59T）,变异等位基因频率（Variant allele frequency, VAF）9.54％，为体细胞突变。诊断PEL，予D-CAG方案化疗，具体为地西他滨20 mg·m^−2^·d^−1^，第1～5天；阿糖胞苷10 mg·m^−2^·次^−1^，每12 h 1次，第1～14天；阿柔比星5 mg·m^−2^·d^−1^，第1～8天；G-CSF 5 µg·kg^−1^·次^−1^，每12 h 1次。治疗中LDH持续>20 000 U/L，合并严重肺部及肠道感染、弥散性血管内凝血，并最终死亡，尚未进行首疗程治疗反应评估，确诊至死亡为1个月余。

例2，男，9月龄余，主因“面色苍白1个月”于2020年4月入院。院外查血常规：HGB 79 g/L，平均红细胞体积（MCV）、平均红细胞血红蛋白量（MCH）轻度降低，外周血涂片红细胞轻度大小不一，中心淡染区扩大，补铁治疗后复查HGB 66 g/L。LDH 3 880 U/L。既往史、个人史及家族史无异常。入院体检：面色苍白，肝肋缘下4 cm，脾肋缘下4 cm。我院首次骨髓穿刺涂片：增生活跃，粒∶红为1∶1，幼稚淋巴细胞占8％，分类不明原始细胞占17.5％。骨髓FCM：CD34^+^细胞占有核细胞比例0.891％，CD71^+^CD45^+^细胞占有核细胞比例10.191％。核型：49，XY,+19,+20,+22[3]/46,XY[17]。融合基因检查发现NFIA::CBFA2T3，基因突变检查检出EPOR:NM_000121,exon8,c.1092_1093del（p.Q365fs*18），VAF 18.9％，为体细胞突变。首次诊断骨髓增生异常肿瘤（MDS），予地西他滨20 mg·m^−2^·d^−1^，第1～5天治疗，联合芦可替尼2.5 mg/次，每12 h 1次，口服。治疗后2周，复查LDH 19 040 U/L，骨髓涂片粒∶红为0.16∶1，红系增生，占53.5％，原始红细胞占50.0％。骨髓FCM：CD45阴性SSC较有核红细胞大的区域可见异常细胞群体，约占有核细胞的30.17％，表达CD36、CD71、CD117，部分表达CD7、CD13、GlyA。修正诊断为PEL。行CLAG+IDA化疗（CL：克拉屈滨，A：大剂量阿糖胞苷，G：G-CSF，IDA：去甲氧柔红霉素）桥接异基因造血干细胞移植（allo-HSCT），干细胞选用HLA 9/10相合非血缘脐带血，预处理方案为白消安4 mg·kg^−1^·d^−1^×4 d+环磷酰胺60 mg·kg^−1^·d^−1^×2 d, +9 d粒系植入，+14 d血小板植入。+20 d、+28 d复查骨髓，形态学完全缓解，FCM-MRD<10^−4^，为完全供者嵌合。+60 d，嵌合度下降，停用免疫抑制剂，予地西他滨治疗，LDH升至2 118 U/L，+74 d复查，骨髓涂片原始红细胞占50％，FCM分析异常红细胞占42.4％，染色体核型：49,XY,del（9）（p22）,+19,+20,+22[15]/48,idem,−4[2]/50,idem,+del（9）（p22）[1]/50,idem,+19[1]/46,XY[1]，嵌合度进一步下降，提示PEL复发，后放弃治疗。+110 d死亡。

例3，男，5岁，为院外诊治后来我院。主因“左侧面部肿物6个月”于2020年7月入院。患儿6个月前于外院发现左侧颌面肿物，行口腔颌面软组织清创术，术后病理为淋巴造血系统肿瘤。外院骨髓形态：增生明显活跃，原始细胞占85.2％；骨髓FCM：B门细胞占45.3％，位于CD45阴性区域，FSC、SSC大，表达CD117、CD36、CD71。后出现发热，伴左眼球突出、闭合不全，行PET-CT示双侧颞极、双侧颞部皮下、双侧眼眶内、左侧颞窝下、双侧颌下区、双侧下颌支内侧、右肺胸膜下及脊柱两旁多发结节状、团块状代谢活跃，颈部双侧Ⅰ～Ⅱ区多个淋巴结、脾脏、中央及外周骨髓弥漫性不均匀性代谢活跃。院外先后行2次面部肿物活检病理会诊考虑淋巴造血系统恶性肿瘤（具体不详）。院外行第2次骨髓穿刺，涂片原始细胞占89.2％，骨髓FCM：B门细胞占78.0％，位于CD45阴性区域，FSC、SSC大，高表达CD117、CD36、CD71。予长春地辛+环磷酰胺+地塞米松化疗效果不佳，疾病进展，双眼突出。院外第3次病理会诊，免疫组织化学染色（IHQ）：CD31（−）、CD61（−）、CD71（+−）、CD42b（−）、TdT（−）、LMO2（+）、Lysozyme（−）、CD117（+）、S100（−），结论倾向于髓系肉瘤。行第3次骨髓穿刺，骨髓FCM：SSC大，CD45阴性区域可见异常细胞群体，约占有核细胞的58.5％，表达CD36、CD71、CD105、CD117。院外予去甲氧柔红霉素+阿糖胞苷（IA）方案2个疗程，联合地塞米松。后颜面部肿块缩小，骨髓形态示增生减低，粒∶红为17.5∶1，红系比例减低，不明细胞占2.4％，FCM检测结果不详。脑脊液FCM可见40.4％与骨髓初诊表型一致的异常细胞，存在中枢神经系统受累，增加鞘内注射次数后复查脑脊液转阴。院外继续予HAE（高三尖杉酯碱+阿糖胞苷+依托泊苷）方案化疗，化疗后未复查骨穿，复查PET-CT示髓外病灶消失，中央及外周骨髓弥漫性代谢活跃，较前未见明显变化。院外继续予IA方案化疗1个疗程，复查骨髓涂片可见54.5％分类不明细胞，FCM检测未见异常细胞，再次出现左侧眼球突出，并逐渐加重，予IA+地塞米松化疗后眼球突出一过性缩小后再次出现，伴双下肢疼痛，遂于我院住院。既往史、个人史及家族史无异常。体检：面色口唇苍白，双眼突出，浅表淋巴结及肝脾未及肿大。我院血常规：WBC 2.11×10^9^/L，ANC 0.64×10^9^/L，HGB 98 g/L，PLT 22×10^9^/L，外周血涂片原始幼稚细胞12％。LDH 13 160 U/L，铁蛋白>40 000 ng/ml。我院骨髓涂片示增生低下，粒∶红为1∶1，原始细胞89％。骨髓FCM分析：异常占有核细胞77.0％，表达CD36、CD71、CD81，部分细胞表达CD13、CD117、GlyA。骨髓染色体核型：50～53,XY,add（1）（p22）,+del（1）（q21）,+6,del（6）（q21）,+7,del（8）（q24）,+19,+20,+4～5mar,inc［cp19］/46,XY[1]。骨髓检出NFIA::RUNX1T1，及以下基因突变：KRAS：NM_033360,exon2,c.G34A（p.G12S），VAF 48.6％，PTPN11:NM_002834,exon3, c.G214A（p.A72T），VAF 42.7％，为体细胞突变。CHEK2:NM_007194,exon11,c.1116dupC（p.K373fs*22），为胚系突变。我院诊断PEL/红系肉瘤（PES），予地西他滨20 mg·m^−2^·d^−1^，阿糖胞苷10 mg·m^−2^·次^−1^，每12 h 1次，阿柔比星10 mg·m^−2^·d^−1^，隔日给药，治疗3 d，患儿双眼突出进行性加重，周身疼痛明显，放弃出院，数天后死亡，首诊至死亡7个月余。

## 讨论与文献复习

红白血病（EL）1912年由Copelli首次报道。1917年，意大利病理学家Di Guglielmo观察到此类疾病为红系前体细胞的肿瘤性增殖并伴随粒系异常[1]。1940年，Dameshek提出将这种疾病命名为Di Guglielmo病，并作为独立于其他AML的新型白血病[2]。同年，Moeschilin建议将此类白血病命名为“EL”。1976年，法国、美国、英国（FAB）合作小组将其归类为AML中的AML-M_6_[2]。之后，随着对该疾病认识的不断深入，其命名也几经演变，2001年，WHO分类将其从形态上分为两个亚型，即红系/髓系白血病（FAB，AML-M_6a_）和PEL（FAB，AML-M_6b_）。2016年，WHO第四版血液及淋巴组织肿瘤分类中，对骨髓有核细胞中红系前体细胞成分≥50％的髓系肿瘤进行了重新定义：在除外重现性遗传学异常和继发因素的情况下，将仅来自于红系（>80％的骨髓细胞为红系，其中≥30％为原始红细胞）的原发未分化或原始红细胞克隆性增殖命名为“PEL”[3]，伴有髓系原始细胞成分的列入MDS或非特指AML分类中。2022年发布的WHO第五版髓系和组织/树突细胞肿瘤分类中，将PEL重新命名为“急性红白血病”（AEL）[4]。为避免概念混淆，本文仍采用PEL进行描述。

PEL可在任何年龄发病。美国基于SEER数据库的研究显示，PEL的年龄分布呈双峰分布，发病高峰主要在70岁以上人群，另一个较小的发病高峰为20岁以下人群，小于18岁的患者约占5％[5]。

PEL骨髓形态以成熟受抑的红系原始细胞增殖为主要特点，缺乏髓系其他原始细胞成分[6]。然而，PEL的发生可能被阻滞在红系分化的任意阶段，甚至包括爆式红系集落形成单位（BFU-E）和红系集落形成单位（CFU-E）阶段，二者从形态上无法帮助确认红系谱系。本研究中的3例患儿均经骨髓穿刺确诊，确诊时仅例1骨髓涂片中红系占比超过80％，例1、2患儿红系前体细胞均以原始红细胞为主。3例患儿均经历多次骨髓穿刺后方获得确诊，说明对于PEL来说，由于取材或病情进展情况不一，仅通过骨髓形态学检查确诊较为困难。

当形态学无法确诊PEL时，需要借助FCM或IHQ方法。在其他淋巴造血系统肿瘤相关染色均阴性的时候，CD43、CD71和E-cadherin染色可作为鉴别诊断PEL的标志[7]。例3行多次骨髓穿刺，形态学可见到大量原始细胞，但FCM标志谱系不特异，未能早期通过FCM确诊。该患儿起病时存在髓外受累，病灶IHQ染色并未做红系特异性标记，曾疑诊“BCOR相关肉瘤”。这可能由于该患儿的肿瘤细胞来源于几乎未分化的原始细胞，无法分辨谱系所致。Lapadat等[8]于2016年报道了与例3类似的一例PEL，为3月龄男婴，以左侧眼球突出起病，组织活检IHQ呈CD45阴性，CD99阳性，电镜下肿瘤细胞胞质中可见多颗粒糖原聚集，符合尤因肉瘤，但由于缺乏尤因肉瘤特异性融合基因，诊断存疑，后经骨髓活检，IHQ标记CD99、CD71阳性，FCM示CD45阴性的大细胞群，CD36、CD13表达阳性，CD34阴性，CD71部分阳性，最终确诊PEL/PES。因此对于形态和FCM、IHQ均不典型的“小圆细胞恶性肿瘤”，需鉴别极为罕见的PEL/PES，可检测包括CD71、CD235、GlyA和E-cadherin在内的红系抗原标志。

本研究中3例患儿均检出NFIA重排/1p31易位。NFIA基因定位在染色体1p31，编码转录因子蛋白Nuclear Factor I-A（NFIA），隶属于NF1家族，是红细胞分化所必需的转录因子。研究发现，NFIA在正常造血过程中呈明显谱系特异性表达模式：在红系中表达增加，但在粒细胞生成中表达被完全抑制[9]。以往个案报道，在儿童PEL中发现1p31易位产生的NFIA::CBFA2T3[9]以及NFIA::RUNX1T1融合基因[10]，因此可认为NFIA重排为儿童PEL的重现性遗传学改变。RUNX1T1又称CBFA2T1，与CBFA2T3均为转录调节因子，参与红系分化及增殖。

为全面了解携带NFIA重排的儿童PEL的临床特征，我们进行了文献复习，检索PubMed、Web of Science和中国知网、万方数据库，检索时间范围从建库至2025年12月，检索关键词包括“pure erythroid leukemia”、“erythroid leukemia”、“erythroblastic sarcoma”、“NFIA rearrangement”、“children”、“pediatric”、“儿童”、“红白血病”、“髓系肉瘤”的中英文组合，共检索到5例伴随NFIA::CBFA2T3或NFIA::RUNX1T1融合基因的儿童PEL个案报道[11]–[14]。此外，尚有2例染色体核型为t（1;16）（p31;q24）的儿童PEL报道[15]–[16]，2例的临床表现与上述病例十分相似，推测也是由于该重排或融合基因致病，将以上病例会同本研究的3例共同分析（**扫描本文首页二维码可查阅附表1**）。除1例未记录外，余9例均行染色体核型检测，7例为复杂核型，其中5例为高二倍体核型，5例合并+19异常改变，提示NFIA重排/1p31易位的PEL容易在干系克隆的基础上衍生旁系克隆。值得注意的是，1例为正常核型，需注意隐匿性核型异常改变。

除融合基因外，6例患者有基因变异的记录。其中2例存在EPOR基因变异，EPOR基因编码正常红细胞发育所必需的促红细胞生成素（EPO）受体，同时作为一种酪氨酸激酶受体，可通过RAS/MAPK通路进行信号传递。2例检出KRAS基因变异。1例检测出LZTR1基因变异。已证实LZTR1基因变异导致的RAS泛素化的缺失可激活RAS/MAPK信号导致多种肿瘤发生[17]。这些基因变异提示RAS/MAPK信号的激活可能在NFIA重排的PEL/PES的发生发展中起作用[15]。动物实验证实NFIA::CBFA2T3融合基因可协同TP53 R248Q突变抑制红系分化的基因表达程序而启动PEL的发生[18]，该TP53突变与成人PEL高度相关[19]，而NFIA::CBFA2T3融合基因目前只在儿童PEL中有报道，未见合并TP53突变的报道。可见该融合基因或者NFIA重排对儿童PEL的致病机制尚需进一步探索，这也表明儿童与成人PEL在发病机制上存在不同。

本组病例及文献复习显示，PEL对传统化疗高度耐药。所有病例均接受化疗，除2例记录不详外，8例采用了AML化疗方案，5例对化疗有反应，可获得骨髓CR，但缓解期较短，3例缓解时间不足3个月。髓外病灶通常对化疗反应较差，1例单纯中枢神经系统（CNS）受累患儿除接受AML化疗方案外还接受了大剂量甲氨蝶呤+门冬酰胺酶化疗，但仍未缓解。2例复发后文献中未记录最终结局，推测结局不良。7例患儿最终因疾病进展死亡，存活时间1～7个月。1例至随访终点时为存活状态，但观察时间仅5个月，根据其他病例推测，可能远期结局不良。1例在首次CR后继续接受HSCT，后期虽CNS复发，但经鞘内注射和放疗后再次获CR，持续存活28个月，为11例中存活时间最长者，提示在诱导首次CR后行HSCT可能增加长期生存机会。

本中心治疗的3例均死亡，3例采用了含有去甲基化药物（HMA）的化疗方案，例2还联合了JAK/STAT通路抑制剂芦可替尼治疗，均未获得满意疗效。例2行CLAG化疗桥接allo-HSCT后获CR，但60 d后复发，疾病持续进展。提示即便HSCT能清除部分肿瘤细胞，但PEL的高度侵袭性和耐药性仍可能导致复发。

PEL发病机制涉及早期前体细胞的分化阻滞和肿瘤细胞增殖，可能与其他AML的双重打击模型类似[20]。在红系谱系分化成熟的过程中，有多种细胞因子、转录因子、染色体修饰因子和微小核糖核酸（microRNA）参与，这些因素的失调可导致红系生成异常性疾病[21]。实验室研究表明表观遗传学改变和microRNA失调对PEL的发病机制至关重要。

近年来有多项应用HMA治疗成人AEL/PEL的研究报道，但其结论存在争议。一项纳入36例成人AEL患者的研究显示，地西他滨治疗AEL患者疗效优于以阿糖胞苷为基础的方案[22]。另有研究报道，HMA一线治疗成人AEL整体反应率46％，CR率为30％[23]，但这两项研究中大部分患者经WHO2016分类重新评估为MDS病例。另一项研究显示AEL和PEL的总体生存率不佳，HMA的应用并未改善其预后[24]。本研究中3例患儿应用HMA后均未获得满意疗效，推测可能由于儿童与成人PEL在发病机制上存在差异有关。

因此，未来儿童原发性PEL的治疗探索，应更多聚焦于靶向关键通路中的新型药物，如溴结构域抑制剂、组蛋白去乙酰化酶抑制剂和microRNA靶向治疗等[6]。此外，鉴于NFIA重排驱动了红系特异性转化，针对NFIA融合蛋白或可能参与驱动肿瘤的其他信号通路，如RAS/MAPK通路（本研究中多例检出KRAS、PTPN11、LZTR1突变）的靶向治疗，是极具潜力的研究方向。另外，一些新型的免疫治疗，如靶向CD71/转铁蛋白受体1的单克隆抗体[25]或嵌合抗原受体T细胞治疗，可能为这类难治性疾病提供新的治疗选择。

## Supplementary Material


